# Flavonoid Analyses and Antimicrobial Activity of Various Parts of *Phaleria macrocarpa* (Scheff.) Boerl Fruit

**DOI:** 10.3390/ijms12063422

**Published:** 2011-05-27

**Authors:** Rudi Hendra, Syahida Ahmad, Aspollah Sukari, M. Yunus Shukor, Ehsan Oskoueian

**Affiliations:** 1 Department of Biochemistry, Faculty of Biotechnology and Biomolecular Sciences, Universiti Putra Malaysia (UPM), 43400 UPM Serdang, Selangor, Malaysia; E-Mails: rootdee2001@yahoo.com (R.H.); yunus@biotech.upm.edu.my (M.Y.S.); 2 Department of Chemistry, Faculty of Mathematic and Natural Sciences, University of Riau, Pekanbaru, Riau, Indonesia; 3 Department of Chemistry, Faculty of Sciences, Universiti Putra Malaysia (UPM), 43400 UPM Serdang, Selangor, Malaysia; E-Mail: aspollah@science.upm.edu.my; 4 Department of Microbiology, Faculty of Biotechnology and Biomolecular Sciences, Universiti Putra Malaysia (UPM), 43400 UPM Serdang, Selangor, Malaysia; E-Mail: ehs424@yahoo.com

**Keywords:** *P. macrocarpa*, flavonoid, antimicrobial activity

## Abstract

*Phaleria macrocarpa* (Scheff.) Boerl (Thymelaceae) is commonly known as ‘Crown of God’, ‘Mahkota Dewa’, and ‘Pau’. It originates from Papua Island, Indonesia and it grows in tropical areas. Empirically, it is potent in treating the hypertensive, diabetic, cancer and diuretic patients. It has a long history of ethnopharmacological usage, and the lack of information about its biological activities led us to investigate the possible biological activities by characterisation of flavonoids and antimicrobial activity of various part of *P. macrocarpa* against pathogenic bacteria and fungi. The results showed that kaempferol, myricetin, naringin, and rutin were the major flavonoids present in the pericarp while naringin and quercetin were found in the mesocarp and seed. Furthermore, the antibacterial activity of different parts of *P. macrocarpa* fruit showed a weak ability to moderate antibacterial activity against pathogenic tested bacteria (inhibition range: 0.93–2.17 cm) at concentration of 0.3 mg/disc. The anti fungi activity was only found in seed extract against *Aspergillus niger* (1.87 cm) at concentration of 0.3 mg/well. From the results obtained, *P. macrocarpa* fruit could be considered as a natural antimicrobial source due to the presence of flavonoid compounds.

## 1. Introduction

*Phaleria macrocarpa* (Scheff.) Boerl (Thymelaceae) is commonly known as ‘Crown of God’, ‘Mahkota Dewa’, and ‘Pau’. It originates from Papua Island, Indonesia and it grows in tropical areas. This plant is one of the most popular medicinal plants in Indonesia. *P. macrocarpa* grows throughout the year in tropical areas reaching a height of around 1–6 m. It is a complete tree (stem, leaves, flower and fruit) and the fruits are eclipse-shaped with a diameter of around 3 cm. The color of the fruit is green before ripening and red when fully ripe [1]. Previous studies on secondary metabolites from *P. macrocarpa* have confirmed the presence of Kaempferol-3-*O*-β-d-glucoside in the fruit and it was found to protect H4IIE rat hepatoma against oxidative stress [2,3]. In addition, Osimi [4] isolated 3 compounds (icariside C3, phalerin, and mangiferin) from the fruit and icariside C3 showed a slow vasorelaxant activity against noradrenaline-induced contraction of isolated rat aorta. Moreover, benzophenone glucoside and 4′-6′-dihyroxy-4-metoxybenzophenone-2-*O*-glucoside were isolated from the fruit and leaves [5,6].

Empirically, it is believed that it has the potential to treat hypertension, diabetes, cancer and diuretic conditions. Natural phytochemicals have been reported to possess a wide range of biological activities including antioxidant, antimicrobial and anti-inflammatory properties [7]. Recently, there has been a growing interest in the investigation and introduction of medicinal plants with various biological activities to the pharmaceutical industries since synthetic drugs have been associated with several side effects on human health. Furthermore, microorganisms indicated a resistance to synthetic antimicrobial agents, which is a serious and immediate concern [8,9]. Due to these facts, the exploration of new alternative medicines derived from plants is required. Flavonoids are classified under phenolic groups in plants which have been known to possess antimicrobial activity [10]. The mechanisms of flavonoids that are antimicrobial can be classified as the inhibition of nucleic acid synthesis, cytoplasmic membrane function, and energy metabolism [11]. There has been a long history of ethnopharmacological usage and the lack of information about *P. macrocarpa* fruit’s biological activities led us to investigate the possible biological activities by the characterization of flavonoids and antimicrobial activity of various parts of this fruit against various pathogenic bacteria and fungi.

## 2. Results and Discussion

### 2.1. Flavonoid Compounds Analyses

**Figure 1:**
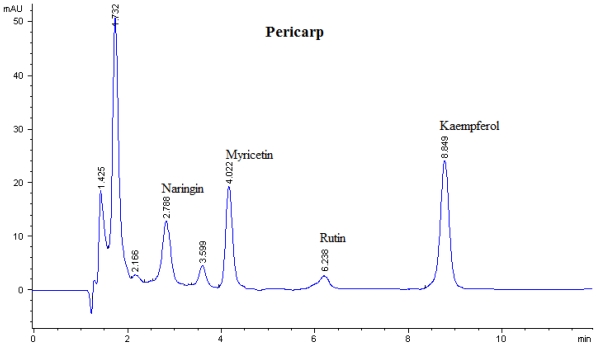
Flavonoids content of pericarp of *P. macrocarpa* analysed by HPLC at the wavelength of 365 nm.

**Figure 2:**
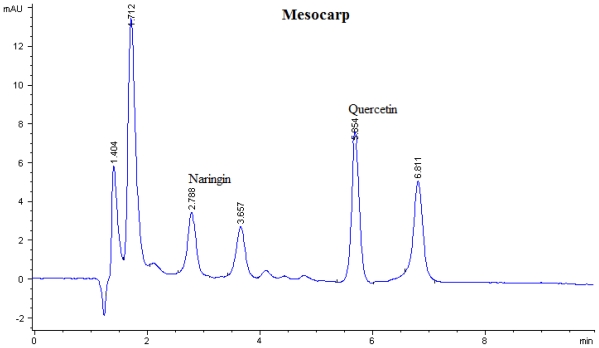
Flavonoids content of mesocarp of *P. macrocarpa* analysed by HPLC at the wavelength of 365 nm.

**Figure 3:**
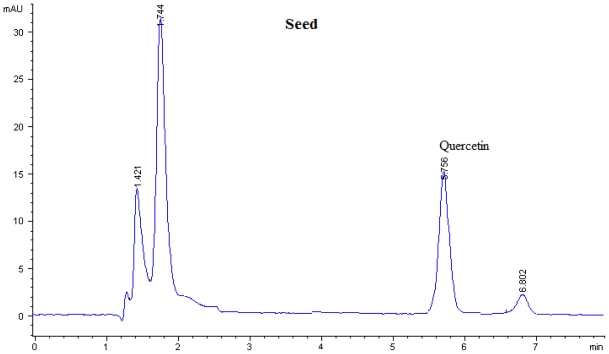
Flavonoids content of seed of *P. macrocarpa* analysed by HPLC at the wavelength of 365 nm.

Flavonoid compounds present in *P. macrocarpa* fruits were analyzed by using Reversed-Phase High Performance Liquid Chromatography (RP-HPLC). The flavonoid compounds have been identified according to their retention times and quantified according to their respective standard calibration curves (Figures 1, 2 and 3). Kaempferol, myricetin, naringin, and rutin were found as flavonoid compounds in pericarp of *P. macrocarpa* fruit (Table 1). The results also confirmed the presence of naringin and quercetin in mesocarp. Moreover, the seed of *P. macrocarpa* fruit contained only quercetin with the value of 0.452 mg/g dried weight (DW).

Among the flavonoids detected, the kaempferol content was significantly (p < 0.05) higher in the pericarp extract (76 μg/g DW). This value was lower than the kaempferol value in Chinese tea leaves (1.56–3.31 mg/g dried leaves) [12] but higher than in strawberries (8 μg/g dried-weight) [13]. This finding was supported by Zhang *et al.* [3] who isolated the kaempferol-3-*O*-β-d-glucoside from *P. macrocarpa* fruit.

Myricetin (59.90 μg/mg DW) located at the pericarp has a lower content compared with blackcurrant (71 μg/mg DW) but higher than blueberry (26 μg/mg DW) [13]. The amount of naringin present at the pericarp and mesocarp (39.00 and 1.90 μg/mg DW) was found to be lower than that of citrus (3.26 mg/g DW) [14]. Furthermore, rutin was detected in pericarp with a value of 17.80 μg/mg DW and it was found to be lower than citrus with a value of 3.26 mg/g DW [14] and *Amaranthus viridis* (58.2 μg/mg DW) [15]. Table 1 shows the concentration of quercetin present in mesocarp and seed with the values of 31.80 and 42.80 μg/mg DW, respectively. These values were found to be lower than the quercetin that is found in onion and garlic which is reported by Crozier *et al.* [16] with value 201 and 227 μg/mg DW.

### 2.2. Antimicrobial Activity

The results obtained from antimicrobial assay are presented in Table 2 at a concentration of 0.3 mg/disc. According to Table 2, the extracts of *P. macrocarpa* fruit showed variable ranges of antimicrobial activities against 8 bacteria (Gram-positive and Gram-negative).

In general, most of the extracts indicated weak to moderate inhibitory activities against the bacteria tested. The extract of *P. macrocarpa* fruits at 0.3 mg/disc showed variable inhibitory activity against all bacteria with inhibition zone diameters ranging from 0.93–2.33 cm, as shown in Table 1. The microorganisms responses were different to the different extracts obtain from various parts of *P. macrocarpa* friut. Pericarp of *P. macrocarpa* fruit showed high inhibition on Gram-positive and Gram-negative bacteria compared to mesocarp, seed and kanamycin (1 μg/disc). In this study, it was shown that all the extracts could give higher inhibition to Gram-positive bacteria compared to Gram-negative bacteria. These results are in agreement with Othman [17] that Gram-negative microorganisms are typically more resistant to antimicrobial agents than Gram-positive bacteria. This has long been explained by the presence of an outer-membrane permeability barrier in Gram-negative bacteria, which limits access of the antimicrobial agents to their targets in the bacterial cells.

Furthermore, all the extracts assayed indicate no activity to weak inhibitory activities against all fungi. Based on Table 1, the antifungal activity of extracts from different parts, pericarp and mesocarp showed no antifungal activity against all fungi tested but seed extract at the concentration of 0.3 mg/well showed antifungal activity against *Aspergilus. niger* with inhibition 1.87 cm. This inhibition was low compared to Amphotericin B (25 μg/well). Interestingly, the tested bacteria and fungi showed different sensitivities to the different extracts of *P. macrocarpa* fruit. These results might be due to the presence of phytochemical in seeds such as phorbol esters. Borris *et al.* [18] mentioned that *Phaleria* sp. contain phorbol esters which are present in the seed of the fruit. Saetae and Suntornsuk stated that phorbol esters were found to be responsible for fungal growth inhibition in the crude extract of *Jatropha curcas* seed cake.

Antimicrobial activities observed in this study might be due to the presence of flavonoid compounds. Extracts of various medicinal plants containing phenolic and flavonoids have been previously reported to possess antimicrobial activity [19,20]. Vaquero *et al.* [21] investigated the properties of gallic, caffeic, vanillic acid, rutin, and quercetin of different wine against pathogenic microorganisms. *Escherichia coli* were the most sensitive bacterium and *Flavobacterium* sp. was resistant against all phenolic compounds tested. The flavonoid analyses (Table 1) revealed the presence of kaempferol, myricetin, naringin, quercetin and rutin in *P. macrocarpa* fruit. The presence of these compounds might contribute to antimicrobial activity of *P. macrocarpa* fruit since Cushnie and Lamb [11] reported that kaempferol, myricetin, naringin, quercetin and rutin have antimicrobial activity against human pathogenic microorganisms with some mechanisms of action such as inhibition of nucleic acid synthesis, cytoplasmic membrane function and energy metabolisms. The antimicrobial activity of the extracts of *P. macrocarpa* fruit might be due to one of the mechanisms of action mentioned above. Antimicrobial activity from pericarp, mesocarp, and seed of *P. macrocarpa* fruit might be due to the presence of kaempferol, myricetin, naringin, quercetin and rutin. Teffo *et al.* [22] isolated four kaempferols from *Dodonaea viscosa* Jacq. var. *angustifolia* leaf extracts and applied them to some human phatogenic bacteria. The results showed that all isolated kaempferols could inhibit growth rate of *Staphylococcus aureus, Enterococcus faecalis, E. coli* and *Pseudomonas aeruginosa*. In addition, Demetzos *et al.* [23] investigated the antimicrobial activity of myricetin and its derivate and the results showed that they could inhibit Gram-positive bacteria compared to Gram-negative. Furthermore, Li, Xu and Mandalari *et al.* [24,25] mentioned that quercetin and naringin have antimicrobial activity.

The results of the present study are quite encouraging as almost all of extracts exhibited antimicrobial activity against most of the pathogens. However, the antimicrobial activity varies widely, depending on the microorganism.

## 3. Experimental Methods

### 3.1. Plant Materials

The fruits of *P. macorcarpa* were obtained from Faculty of Mathematic and Natural Sciences, University of Riau, Riau province, Indonesia. The plant species was identified by the laboratory of Plant Taxonomy staff at Herbarium Bogoriense, Bogor, Indonesia. The voucher specimen (SA1611/2008) was deposited at Herbarium Bogoriense, Bogor, Indonesia. The fruits were washed and separated into pericarp, mesocarp and seed. Those parts were air-dried for 7 days and kept for further analyses.

### 3.2. Extraction

The extractions of *P. macrocarpa* were carried out based on Crozier *et al.* [16] with some modification. Air-dried powders of each part of *P. macrocarpa* (0.5 g) were weighed and placed into a 100 mL conical flask. 40 mL of methanol was added, followed by 10 mL and 6 M HCL solution. The mixture was stirred using a magnetic stirrer. The mixture was placed in a sample flask (250 mL), attached to reflux for 2 h at 90 °C, then the mixture was filtered using Whatman No.1 filter paper (Whatman, England), and taken to dryness by using a vacuumed Rotary Evaporator (Buchii, Switzerland) at 40 °C.

### 3.3. Determination of Flavonoid Compounds by HPLC

The flavonoid compounds of different parts of *P. macrocarpa* fruit were quantitatively measured by a reversed-phase HPLC technique based on the method described by Crozier *et al.* [16]. Flavonoid compounds standards consisted of quercetin, rutin, myricetin, kaempferol, naringin, apigenin, and luteolin. an aliquot of sample extract was loaded on a high-performance liquid chromatography (HPLC) Agilent-1200 series instrument equipped with a UV-Vis photodiode array (DAD) detector, binary pump, vacuum degasser, auto sampler and analytical column (Nova-Pak^®^ C18 60Å 4 μm 3.9 × 150 mm, Waters, NANPA, MA, USA). The mobile phase comprises deionized water and acetonitrile. The pH of water was adjusted to 2.5 with trifluoroacetic acid. The flavonoid compounds were detected at 365 nm. The column was equilibrated by 100% solvent A (15% acetonitrile) then the ratio of solvent B (35% acetonitrile) was increased to 100% in 20 min. This ratio was maintained for the rest of the analysis with a flow rate of 1 mL/min.

### 3.4. Antimicrobial Activity

#### 3.4.1. Bacteria and Fungi Cultures

The Gram-negative and -positive bacteria (Bacillus cereus, Bacillus subtilis, Enterobacter aerogenes, E. coli, Klebsiella pneumonie, Micrococcus luteus, P. aeruginosa, and S. aureus), fungi (A. niger, Fusarium oxysporum, Ganoderma lucidum, and Mucor indicus) were all purchased from the Institute of Malaysian Research (IMR) and maintained in the Department of Microbiology, Faculty of Biotechnology and Biomolecular Sciences, Universiti Putra Malaysia.

#### 3.4.2. Bacterial Susceptibility Testing

The antibacterial assay of *P. macrocarpa* fruit extracts was carried out by the disc diffusion method [26]. All the microorganisms mentioned above were incubated at 37 °C for 24 h by inoculation into nutrient broth. The culture suspensions were prepared and adjusted to approximately 10^5^ c.f.u. of bacteria/mL. One hundred microliters of the inoculate were spread over plates containing sterile nutrient agar. Paper filter discs (6 mm) impregnated with 10 μL (0.30 mg/disc) of each extract were placed on the surface of the media. The plates were left for 30 min at room temperature to allow the diffusion of the extracts and incubated at 37 °C for 24 h. Finally, the inhibition zone around the disc was measured. Kanamycin (1 μg/disc) was also included in the test as a reference control to evaluate the susceptibility of tested strains. The experiments were run in triplicate.

#### 3.4.3. Fungi Susceptibility Testing

The antfungal assays of *Phaleria macrocarpa* fruit extracts were carried out by the agar well diffusion assay [27]. Briefly, a suspension of the tested fungi was prepared (10^5^ spore/mL), added 100 μL into agar plate and dispensed uniformly in the surface of the agar plate. The small wells were cut in the agar plate using a cork borer (6 mm). A fixed volume of extracts (0.3 mg/well) and (25 μg/well) Amphotericin B (PAA Lab., Cölbe, Germany) were loaded in the wells. The plates were incubated at 29 °C for 72 h and the diameter of the inhibition zone around each well was recorded.

## 4. Conclusions

This study showed that *P. macrocarpa* fruit contained kaempferol, myricetin, naringin, and rutin as flavonoids. Moreover pericarp, mesocarp, and seed extract were potent as antimicrobial agents. The presence of flavonoids could contribute to its antimicrobial activities. It is suggested that *P. macrocarpa* fruit could be considered as source of antimicrobial agent which might be applied in pharmaceutical and cosmetic products.

## Figures and Tables

**Table 1:** Contents of flavonoids compounds in pericarp, mesocarp, and seed of *P. macrocarpa* fruit.

Sample	Flavonoid contents (μg/g DW)						
	Apigenin	Kaempferol	Luteolin	Myricetin	Naringin	Quercetin	Rutin
Pericarp	-	76.00 ± 0.003	-	59.90 ± 0.001	39.80 ± 0.001	-	17.80 ± 0.001
Mesocarp	-	-	-	-	1.90	31.80 ± 0.002	-
Seed	-	-	-	-	-	45.20 ± 0.003	-

-Not detected; analysis were done in triplicate.

**Table 2:** Antimicrobial activity of extracts obtained from various parts of *P. macrocarpa* fruit.

Microorganisms	Inhibition zone (cm)*				
	Pericarp	Mesocarp	Seed	Kanamycin (1 μg/disc)	Amphotericin B (25 μg/well)
**Gram-Positive Bacteria**					
*B. cereus*	1.73	1.53	1.40	1.63	-
*B. subtilis*	2.33	2.00	1.83	1.33	-
*M. luteus*	1.57	1.47	1.37	1.35	-
*S. aureus*	1.53	1.73	1.40	1.35	-
Average	1.79	1.68	1.50	1.42	-
**Gram-Negative Bacteria**					
*E. aerogenes*	1.50	1.30	1.20	1.55	-
*E. coli*	2.17	1.47	1.47	1.73	-
*K. pnuomoniae*	1.23	1.10	0.97	1.5	-
*P. aeruginosa*	1.40	1.17	0.93	1	-
Average	1.56	1.26	1.14	1.45	-
**Fungi**					
*A. nige*	0	0	1.87	-	2.70
*F. oxysporum*	0	0	0	-	1.80
*G. lucidum*	0	0	0	-	0.40
*M. indicus*	0	0	0	-	1.20
	0	0	0.47	-	1.53 ± 0.97

* Inhibition zone in diameter (cm); 0: no inhibition; analyses were done in triplicate.
